# Antimutagenic and Synergistic Cytotoxic Effect of Cisplatin and Honey Bee Venom on 4T1 Invasive Mammary Carcinoma Cell Line

**DOI:** 10.1155/2019/7581318

**Published:** 2019-01-29

**Authors:** Faranak Shiassi Arani, Latifeh Karimzadeh, Seyed Mohammad Ghafoori, Mohammad Nabiuni

**Affiliations:** ^1^Department of Animal Biology, Faculty of Biological Science, Kharazmi University, Tehran, Iran; ^2^School of Biology, College of Science, University of Tehran, Tehran, Iran; ^3^Department of Genetics, Islamic Azad University, Tehran Medical Branch, Tehran, Iran; ^4^Department of Cell and Molecular Biology, Faculty of Biological Sciences, Kharazmi University, Tehran, Iran

## Abstract

**Introduction:**

Honey bee venom (HBV) has various biological activities such as the inhibitory effect on several types of cancer. Cisplatin is an old and potent drug to treat most of the cancers. Our aim in the present study was to determine antimutagenic and cytotoxic effects of HBV on mammary carcinoma, exclusively and in combination with cisplatin.

**Methods:**

In this study, 4T1 cell line was cultured in RPMI-1640 with 10% fetal bovine serum (FBS), at 37°C in humidified CO_2_ incubator. The cell viabilities were examined by the MTT assay. Also, HBV was screened‏ for its antimutagenic activity via the Ames test. The results were assessed by SPSS software version 19 and one-way ANOVA method considering *p* < 0.05 level of significance.

**Results:**

The results showed that 6 mg/ml of HBV, 20 *μ*g/ml of cisplatin, and 6 mg/ml HBV with 10 *μ*g/ml cisplatin could induce approximately 50% of 4T1 cell death. The concentration 7 mg/ml of HBV with of 62.76% inhibitory rate showed the highest antimutagenic activity in comparison with other treatment groups.

**Conclusions:**

The MTT assay demonstrated that HBV and cisplatin could cause cell death in a dose-dependent manner. The cytotoxic effect of cisplatin also promoted by HBV. Ames test outcomes indicated that HBV could act as a significant mutagenic agent. The antimutagenic activity of HBV was increased considerably in the presence of S9 mix. Therefore, our findings have revealed that HBV can enhance the cytotoxic effect of cisplatin drug and its cancer-preventing effects.

## 1. Introduction

Breast carcinoma is a common cancer and the most common malignancy among women in the industrialized countries for the last decades. Breast cancer originates from the breast tissue and uncontrolled growth of the inner lining of the milk ducts or lobules [1]. Breast cancer is the most commonly diagnosed cancer and the primary cause of cancer death among worldwide females [2]. Several studies have shown that breast carcinoma mostly occurs in similar structures of the breast, and these structures are the origin site of ductal carcinoma. Approximately 1-2 years after the onset of the first menstrual period, lobule formation is started. There is a gradual process for mammary gland, for which it needs several years. Parous women, particularly women who have full-term pregnancy experience at young age, have full lobular differentiation in breast structure [3]. The most risk factors for breast cancer have been estimated and studied such as diet, oral contraception, and postmenopausal substituent treatment with estrogen, breast irradiation, and environment [4].

The 4T1 mouse mammary tumour cell line is a model of breast cancer, which is able to metastasize to site affected in human breast cancer. In 1983, Fred Miller and coworkers isolated the 4T1 mammary mouse carcinoma cell line from BALB/c mammary tumour. This cell line has the potential to metastasize to bone and several organs affected by breast cancer consist of the lung, liver, and brain. Hence in recent years, usage of this cell line has increased [5].

One of the most important causes of spontaneous breast cancer is genetic mutations. The molecular studies of mammary tumours indicated that the amplification of oncogenic genes such as erb-B1, erb-B2, c-myc, and int-2 is common [6]. About 5–10% of all breast cancer cases are hereditary breast cancer. BRCA1, tumour suppressor gene, is mutated in the most hereditary breast and ovarian cancer [7].

Recently, the treatment of cancer becomes a global concern. The various types of cancer treatment ways, such as surgery, the anticancer drug (chemotherapy), irradiation, hormone therapy, and nutritional supplementation are used. Chemotherapy is a systematic therapy in which all of the body cells are exposed to chemotropic agents [8].

Using metals as medicine backs to 5000 years ago. The investigations on minerals resulted in developing modern medicine with metal components for the treatment of some diseases such as cancer. Cisplatin (*cis*-dichlorodiammineplatinum (II)) is the first stage of chemotherapy for most cancers, including testicular, ovarian, cervical, small lung-cell, and also breast cancer [9].

The cytotoxic effect of cisplatin depends on dosage, and its high dosage has improved effects on cancerous cells. However, the application of cisplatin is subject to some restrictions as it has several potential side effects, special nephrotoxicity, and neurotoxicity effects [10].

Honey bee venom (HBV) is an active product which is produced by the venom glands associated with the sting apparatus of honey bee workers and their queen. The history of apitherapy returns to 3000 to 5000 years ago [11]. HBV is a complex mixture of active enzymes, peptides, and amines. Its most important components are melittin and phospholipase A2, adolapin, and mast cell degranulating peptide. Several in vitro and in vivo studies revealed that HBV shows anti-inflammatory, cytotoxic, and antibacterial effects, and also it can cause a severe allergic reaction [12]. HBV as an old medicine has been used to treat arthritis, rheumatism, back pain, cancerous tumours, and skin diseases [13].

According to anticancer effects of HBV, the aim of this study was to investigate the cytotoxic effect of honey bee venom on the 4T1 cell line, lonely and in combination with Cisplatin. Also, antimutagenic activity and anti-cancerous effects of bee venom were studied by Ames test.

## 2. Materials and Methods

### 2.1. Cell Culture

The mouse mammary carcinoma 4T1 cell line was purchased from cell bank of Pasteur Institute of Iran. Cells were cultured in RPMI-1640 (Gibco-Invitrogen) with 10% fetal bovine serum (FBS) (Gibco-Invitrogen) and antibiotics (100 U/ml penicillin and 100 mg/ml streptomycin) at 37°C in a 5% CO_2_ and 95% O_2_ humidified incubator. The culture medium was changed every 24 h.

### 2.2. HBV Preparation

Honey bee venom was mustered from *Apis mellifera* using an electric shocker apparatus composed of a shocker and collector unit. The shocker unit produces a light electric shock once every few seconds. Honey bees were incited through light electric shock and sting. The collector device is a network of wires with small gaps and a glass plane between them. Every 25 minutes, the shocker unit turned off, and the dried bee venom material on the collector panel was collected by scraping.

HBV was stored in powder form at −20°C and dark condition. The main stock solution of HBV was prepared with 1 mg of HBV and 1 ml phosphate-buffered saline (PBS). In the end, to obtain a homogenous and sterile solution, the solution was passed through a 0.2 *µ*m filter. For every assay, this solution was prepared freshly. For the Ames assay, the concentration of the primary stock solution was 10 mg/ml (HBV + PBS) and other interested concentrations were obtained by making dilution from the main solution.

### 2.3. Cisplatin Preparation

Cisplatin was purchased from Sobhan Oncology Company of Iran with 50 mg/ml concentration, and it was stored in the 4°C and dark situation. Interested concentrations were obtained by diluted the main solution.

### 2.4. MTT Assay

The MTT assay is one of the basis reductions of yellow MTT-dimethylthiazol diphenyl tetrazolium bromide (tetrazole) to the purple formazan crystal by mitochondrial dehydrogenase in living cells. Adherent 4T1 cells were trypsinized by trypsin-EDTA 0.25% (Gibco-Invitrogen). Then, the cells seeded in 24-well plate and cultured overnight in order to fully adhere the cells to the plate. The 4T1 cells were treated with different concentrations of honey bee venom as follows: zero as the control, 2, 4, 6, 8, and 10 mg/ml, and cisplatin, 0 as a control, 5, 10, 15, 20, 25, and 30 *μ*g/ml, and also cisplatin and HBV together, 0 as control, 2 + 10, 4 + 10, 6 + 10, 8 + 10, and 10 + 10 mg/ml, for 24 h. Cell viability was measured using the MTT assay (Sigma-America). MTT solution was prepared (5 mg MTT powder in 1 ml PBS), and then it was filtered via 0.2 *µ*m micropore filter. After 24 h incubation of treated cells, 50 ml MTT solution was added to each well, and the plate was incubated at 37°C for 4 hours and dark situation. Subsequently, the supernatant liquid was removed, 1 ml dimethylsulfoxide (DMSO) (Merck, Germany) was added to each well, and the plate was kept at room temperature for 15 minutes. Finally, the absorbance was measured at 570 nm wavelength by a spectrophotometer (Milton Roy-Spectronic 2ID- America). The viability percent was calculated as follows:

(1) $$\begin{array}{c} \text{viability percent}=\;\frac{\text{optical density of experimental group}}{\text{optical density of control group}}\times 100. \end{array}$$

### 2.5. MIC Assay

To determine the minimum inhibitory concentration of honey bee venom, the MIC assay was performed. Salmonella TA100 suspension was cultured in nutrient broth medium and justified by comparison with 0.5 Mc-Farland turbidity standard tube (1.5 × 108 organisms/ml). The main stock solution of HBV with 10 mg/ml concentration was prepared. Then the main stock was diluted, and 1 mg/ml to 10 mg/ml concentrations were obtained. Finally, each tube received a specific level of HBV. Test tubes were incubated at 37°C for 24 h. Distilled water was used as a negative control. The growth of bacteria in control and test tubes were investigated after 24 h.

### 2.6. Ames Test

The Ames test is designed for analysis of mutagenic and antimutagenic factors [14]. Salmonella TA100, which is used in this test, has various mutations in histidine operon genes. Therefore, in the absence of histidine, the bacteria are not able to grow and create a colony. In the presence of mutagen factors, reverse mutation is occurring, so the bacteria are able to grow and form colonies [15]. Histidine-dependent strain of *Salmonella typhimurium* TA100 used for the Ames test. *Salmonella typhimurium* TA100 developed by Dr Ames of the University of California, Berkeley, USA, was cultured in nutrient broth (Sigma, America). The bacterial suspension was prepared 1-2 × 10^9^ cells/ml fresh cultures.

To prepare of rat microsomal liver enzyme (S9), the mature male rats (about 200 g body weights) were deprived of food for 48 h to achieve high-level hepatic enzymes. Then, the rats were killed and the livers were removed. After washing with PBS solution, the livers were cut into small pieces and homogenized by 1M KCl solution. Finally, this solution was centrifuged for 10 min at 8700 rpm. The supernatant was isolated and stored at −80°C.

- Test groups: 100 *μ*l bacteria suspension, 100 *μ*l histidine-biotin solution (24 mg biotin + 31 mg histidine in 250 ml distilled water), and 100 *μ*l sodium azide solution (10 ml distilled water plus 0.015 g sodium azide) were added to the test tube containing Top Agar (0.6 g agar plus 0.5 g NaCl plus 100 ml distilled water), and finally the test tubes were incubated with 1–7 mg/ml concentrations of HBV.
- Positive control: 100 *μ*l bacteria suspension, 100 *μ*l histidine-biotin solution, and 100 *μ*l sodium azide solution were combined to a tube contain Top Agar.
- Negative control: 100 *μ*l bacteria suspension, 100 *μ*l histidine-biotin solution, and 100 *μ*l distilled water were combined to a test tube contain Top Agar.

Finally, the content of these tubes after 3-second shaking was distributed on the top of the minimum medium of glucose agar (% 40 glucose). The plates were incubated at 37°C for 48 hours. All of these antimutagenic assays were performed in the absence and presence of S9, and for each test, three repeats were considered. Finally, reversed colonies were counted and inhibition percentage was calculated by this formula:

(2) $$\begin{array}{c} \text{inhibition percentage}=\;\left[\frac{1-T}{M}\right]\times 100\text{,} \end{array}$$

where *T* is the number of revertants per plate in the presence of mutagen and test sample and *M* is the number of revertants per plate in the positive control.

### 2.7. Statistical Analysis

The results were assessed by the one-way ANOVA method and also in combination with the Tukey test for pairwise comparison. *p* values less than 0.05 were considered significant. Statistical analysis was performed by SPSS 22.0, and the charts were drawn by Excel software.

## 3. Results and Discussion

### 3.1. MTT Assay

**Figure 1:**
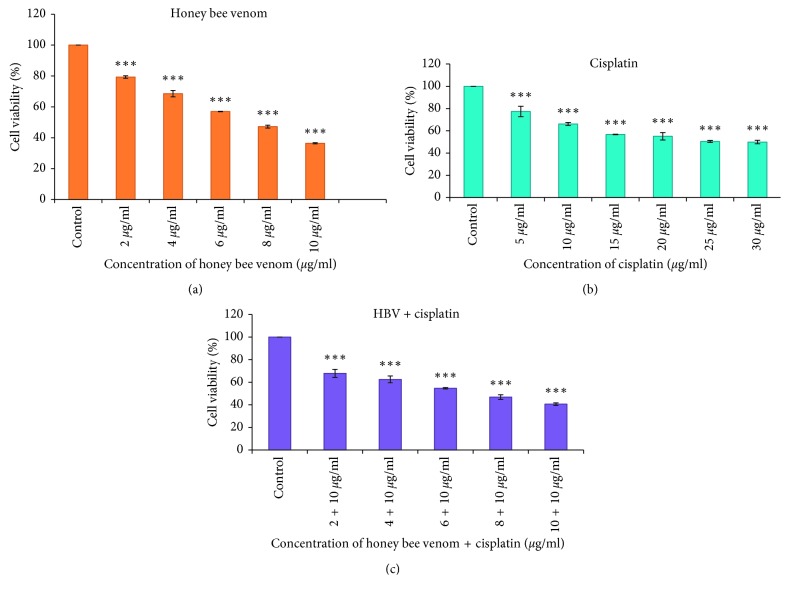
The cell viability percentages of a treated 4T1 cell line with various concentrations of HBV (a), cisplatin (b), and HBV/cisplatin (c) after 24 h by MTT staining (mean ± SEM, ^*∗∗∗*^*p* < 0.001). HBV: honey bee venom.

For investigating the cytotoxic effect of HBV and cisplatin on the 4T1 cell line, the cells were treated with various concentrations of HBV and cisplatin alone and in combination (HBV/cisplatin). Also, to determine cell viability, the MTT assay was performed. The MTT assay revealed that cisplatin and HBV have a cytotoxic effect on 4T1 cell line, and they can reduce the cell viability in a dose-dependent manner. As shown in Figure 1(a), by increasing of HBV concentrations, the cell viability has been reduced. Also, the treated group in comparison with the control group has a significant reduction of viability in a dose-dependent manner (*p* < 0.05) (Figure 1(a)).

On the other hand, cisplatin has a cytotoxic effect on the 4T1 cell line. High concentrations of cisplatin have shown more effective cytotoxicity in comparison with the control group (*p* < 0.001) (Figure 1(b)). Combination treatment of HBV and cisplatin on the 4T1 cell line showed that HBV could promote the cytotoxic effect of cisplatin in a dose-dependent manner (Figure 1(c)). Treatment with 6 *μ*g/ml HBV and 25 *μ*g/ml with cisplatin for 24 h can cause an approximately 50% 4T1 cell death. In combination, cisplatin and HBV, 6 *μ*g/ml + 10 *μ*g/ml can cause about 50% cell death.

### 3.2. MIC Assay

The minimum inhibitory concentration assay for serial dilution concentrations of HBV was performed. The survey of results indicated that HBV can cause death in salmonella TA100 with dosages more than 8 mg/ml. The tubes with 1 mg/ml to 7 mg/ml concentrations showed turbidity, so the bacteria were able to grow. Hence, the MIC of HBV on *Salmonella* TA100 was determined to be 8 mg/ml.

### 3.3. Ames Assay

**Figure 2:**
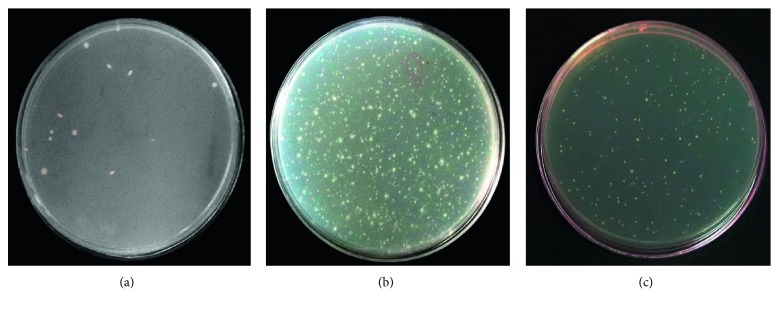
The revertants colonies in the negative test (a), positive test (b), and 7 mg/ml concentration of HBV (c).

**Figure 3:**
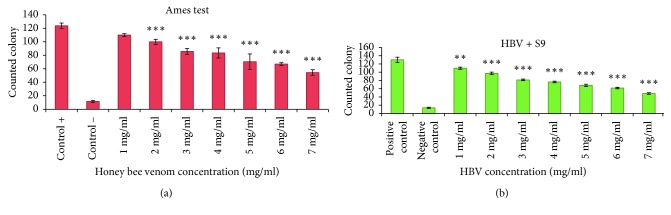
Reverted *Salmonella* TA100 colonies counts in compression with a positive control group with (a) and without S9 (b) by the Ames test (mean ± SEM, ^*∗∗∗*^*p* < 0.001, ^*∗∗*^*p* < 0.01).

**Figure 4:**
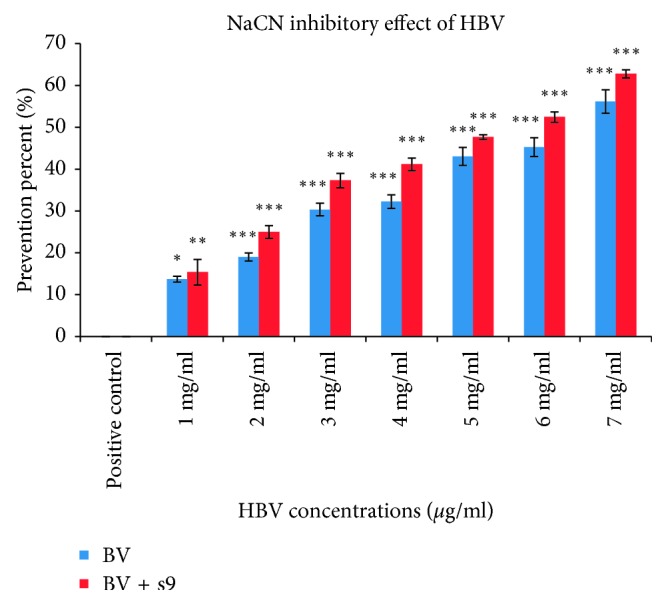
Sodium azide inhibitory effect of HBV in comparison with the positive control group with and without S9 fraction by the Ames test. S9 fraction promoted the prevention effect in a dose-dependent manner (mean ± SEM, ^*∗∗∗*^*p* < 0.001, ^*∗∗*^*p* < 0.01, ^*∗*^*p* < 0.05).

To examine the antimutagenic and anticancerous activities of HBV, the Ames test was performed with 1 to 7 mg/ml concentrations of HBV (less than MIC) in the presence and absence of S9 fraction. After 48 hours, reversed colonies were counted (Figure 2). The plates with different concentrations of HBV have shown reduced colonies in a dose-dependent manner. Comparison between the test and positive control groups has demonstrated significant differences (*p* < 0.001) (Figure 3(a)). Also, the Ames assay was performed in the presence of S9, and the result indicated that antimutagenic activity was improved with S9 (Figure 3(b)). The inhibition percentages of HBV in the presence and absence of S9 were obtained, 62.76 and 56.17 (Figure 4).

## 4. Discussion

Breast cancer is the most prevalent cancer among women of developed countries, and its incidence has been expanding worldwide [16]. The cytotoxic chemotherapy is used to cure the early and late stages of most cancers in the recent decade [17]. In the 1970s, findings about the anticancer effect of Cisplatin lead to a revolution in the clinical chemotropic agent [18]. Our objectives included examining the cytotoxic and antimutagenic effects of HBV and cisplatin on the mouse mammary carcinoma 4T1 cell.

Our results revealed the cytotoxic activity of cisplatin on 4T1 cell line. Determined IC50 value for cisplatin was 25 *μ*g/ml after 24 hr. Cisplatin, *cis*-diamino-dichloro-platinum (II) (CDDP), a complex containing a central atom of platinum to which two ammonium ions and two chlorine ions are bonded in the *cis* position with respect to the horizontal plane of the molecule [19].

Positively charged metals are able to bind to the negatively charged molecules such as protein and nucleic acid [20]. The evidence suggests that the membrane proteins such as the copper transporter 1 (CTR1) accumulate cisplatin in the cells. Meanwhile, replacement of water molecules with chloride ligands activates the cisplatin molecule. The equated forms of cisplatin can bind to DNA at the N7 position of purine bases and form primarily 1,2-intrastrand adducts between adjacent guanosine residues [21]. This cross-linking with DNA and the resulting DNA bending disrupt the replication and transcription process. Hence, the cell cycle is arrested or the apoptosis process will be activated [18]. Cisplatin and other platinum complexes such as carboplatin and oxaliplatin are more useful and effective for treating the most of cancers; however, these components have several severe side effects which affect other healthy organs. Therefore, these side effects result in some limitations of using these chemodrugs in cancer treatment [21].

Most of the new small molecules discovered in cancer treatment are natural products and their derivatives [22]. HBV as a natural product has been used for medicinal applications for thousands of years. HBV contains at least 18 pharmacologically active components, including melittin, phospholipase A2, histamine, and adolapin. The outcomes of various studies on the biological activity of HBV suggest that it is highly effective in several diseases such as arthritis, MS, and back pain [11].

In the present experiment, the concentration of HBV that inhibited growth 4T1 cell line by 50% after 24 hr (IC50) was 6 *μ*g/ml. Our result is different in comparison with the lethal dosage in other cell lines. As illustrated, this level was 1.43 g/ml for mammary carcinoma MCa [23]), 2 *μ*g/ml for human leukemic U937 cell line [12]), 8 *μ*g/ml for A2780cp [21]), 2 *µ*g/ml for human melanoma A2058 cell lines [24], and 10 *µ*g/ml‏ for the human lung cancer NCI-H1299 cell line [25].

Hait et al. [26] demonstrated that melittin is one of the most potent inhibitors of calmodulin activity and also a potent inhibitor of cell growth and clonogenicity. The calcium binding protein, which is named calmodulin, plays a vital role in cellular proliferation [26]. In 2003, Oroli et al. investigated the effect of HBV on MCa in both in vitro and in vivo environments. His results showed that HBV exerts direct (inhibition of calmodulin and prevention of cell growth) and indirect (the stimulation of macrophages and cytotoxic T lymphocytes) effect on MCa tumour cells [27]. Furthermore, Moon et al. [12] studied the key regulators in HBV-induced apoptosis in human leukemic U937 cells. Their outcomes confirmed that HBV inhibits cell proliferation via inducing apoptosis in U937 cells through downregulation of Bcl-2 and upregulation of caspase-3. They demonstrated that HBV increases Fas/Fas ligand levels and decreases Cox-2 and hTERT [12]. According to their study, it can be presumed that HBV induces apoptosis through the extrinsic pathway.

In 2008, Siu-Wan Ip indicated that HBV induces the mitochondria-dependent pathway of apoptosis in human breast cancer MCF7 cells. Their results confirmed that HBV induces DNA strand breaks and promoted P53 and P21 factors. HBV also affects the ratio of Bax/Bcl-2 level, leading to releasing cytochrome c and finally triggering of the mitochondrial apoptosis pathway [28].

Accordingly, our findings are in agreement with other reports about the cytotoxic effect of HBV on cancerous cells. While HBV is probably targeting the DNA molecule and also inhibits calmodulin protein, it is probable that HBV exerts its cytotoxic and growth prevention effects on 4T1 cell line through the intrinsic/extrinsic apoptosis pathway or cell cycle arrest.

Our findings revealed that the cytotoxic effect of cisplatin is risen by a combination of nonlethal concentrations of HBV. In 2009, Orsolic investigated the cytotoxic effect of HBV in combination with Bleomycin in Hela and V79 cell lines. The nonlethal dosage of HBV with bleomycin cause increase death cell in a dose-depended manner. He suggested that HBV inhibits DNA repair, and this may be the mechanism by which it increases bleomycin lethality and inhibits recovery from bleomycin-induced damage [23].

On the other hand, Gajski and Garaj-Vrhovac [29] demonstrated that HBV induces single- and double-strand DNA breaks in human lymphocytes [29]. According to these findings, since cisplatin targets the DNA molecule, it is probable that HBV is able to promote the cytotoxic effect of cisplatin via this mechanism. Our data in the present investigation are similar to Alizadeh's study on the investigation of the synergistic effect of BV and cisplatin on human ovarian cancer cell line A2780cp. He showed that HBV boosts the cytotoxic effect of cisplatin [21].

A mutation, which is a natural process that changes a DNA sequence, is one of the most important causes of cancer. Also, changes in the structure of chromosomes have a critical role in creating most of malignancies [30]. In this study, HBV inhibited reverse mutation in *Salmonella typhimurium* TA100 in a dose-dependent manner. According to Ames theory, the number of revertants colony on the positive control plate (with carcinogen substrate) should be 2 times more than test plates. Also, mutagen prevention percentage contains three classes including inhibitory percent more than 40% which shows high prevention potential, between 25 and 40% which shows medium potential and less than 25% which shows negative prevention [30].

The outcomes of the present investigation showed that the number of reversed colonies in the treated plate with 7 mg/ml concentration of HBV (with S9 and without S9) is less than half of colonies in positive control plate. On the other hand, the inhibitory percentage for 5 mg/ml, 6 mg/ml, and 7 mg/ml concentrations of HBV was 43.05%, 45.26%, and 56.17% in the absence of S9, respectively. Furthermore, the S9 solution improved the HBV inhibitory effect. The S9 fraction is prepared from the liver of rats, and it contains a hepatic enzyme which effectively converts bioactive promutagens to mutagens.

This metabolic activation of mutagens is considered a vital step for carcinogenesis because most of the carcinogen must enzymatically transform to electrophilic specious. This activated mutagen can covalently bind to DNA molecule leading to mutation [31, 32]. Other studies were performed to investigate the antimutagenic potential of some natural products. In 2011, Ghazali et al. confirmed that extract of *M. speciosa* indicates antimutagenic activity [33]. Also, Issazadeh and Aliabadi [34] concluded that olive leaf shows antimutagenic and anticarcinogenic effects [18]. Also, our data demonstrated that, in the presence of S9 fraction, the mutagen prevention percentage of HBV was promoted. Since S9 fraction activates sodium azide mutagen, it is likely that HBV prevents the mutagenic effects of sodium azide. Finally, our result is in agreement with previous studies and its interpretation confirmed that HBV has antimutagenic and anticancerous effect in a dose-independent manner.

## 5. Conclusions

According to our results in the present research, we assessed that HBV as a natural product has cytotoxic effects on the mouse mammary carcinoma 4T1 cell line. Cisplatin as a chemotropic drug to cure several types of cancer has cytotoxic effects on the 4T1 cell line. However, in combination with HBV, its cytotoxic effect is promoted, and it can be more effective in nonlethal dosages. Furthermore, antimutagenic and anticancer activities of HBV were seen in the presence of the S9 metabolic activation system in all concentrations of HBV. It will be an excellent perspective to innovate approaches to prevent and treat some features of cancer.

## Data Availability

The data used to support the findings of this study are included within the article.

## Abbreviations

Cisplatin: *cis*-Diamino-dichloro-platinum (II)
CTR1: Copper transporter 1
DMSO: Dimethylsulfoxide
FBS: Fetal Bovine Serum
HBV: Honey Bee Venom
KCl: Potassium Chloride
MIC: Minimum Inhibitory Concentration
MTT: Dimethylthiazol Diphenyl Tetrazolium Bromide
NaCl: Sodium chloride
PBS: Phosphate Buffer Saline
S9: Supernatant fraction.
